# Aneurysm and Neurocysticercosis: Casual or Causal Relationship? Case Report and Review of the Literature

**DOI:** 10.1155/2011/782496

**Published:** 2011-10-30

**Authors:** Svetlana Agapejev, João Luiz Parra-Marinello, Rodrigo Bazan, Anete Kinumi Ueda, Marco Antonio Zanini

**Affiliations:** ^1^Division of Neurology, Faculty of Medicine, São Paulo State University (UNESP), 18618-970 Botucatu, SP, Brazil; ^2^Division of Neurosurgery, Faculty of Medicine, São Paulo State University (UNESP), 18618-970 Botucatu, SP, Brazil; ^3^Department of Pathology, Faculty of Medicine, São Paulo State University (UNESP), 18618-970 Botucatu, SP, Brazil

## Abstract

Four cases of suggestive inflammatory aneurysms in patients with neurocysticercosis have been described. We report a case of a 49-year-old woman who presented with subarachnoid haemorrhage from a right middle cerebral artery bifurcation aneurysm and had a casual relationship with neurocysticercosis. At surgery, a viable cysticercus without signs of inflammation or thickened leptomeninges was found in the distal position of the aneurysm. Postoperatively, the patient received albendazole and dextrochlorpheniramine. In the subsequent three years, the patient was asymptomatic and took drugs to prevent convulsion and arterial hypertension. The relationship between NCC and the presence of cerebral aneurysm is discussed.

## 1. Introduction

Cysticercosis is the infection of humans and swine by the metacestode larval form of the parasite *Taenia solium* caused by ingestion of *Taenia solium* eggs dispersed by a human *T. solium* tapeworm carrier, producing reactions either in the tissue around the parasites and far from them. Anyway, it is possible to say that transmission of taeniosis/cysticercosis dyad occurs from swine to man, from man to swine, and from man to man. In other words, where there is animal cysticercosis, man is always present and responsible for its dissemination. The increase of migration, immigration, tourism, and globalization strengthen this dissemination [1]. The two basic mechanisms of action, which determine the clinical picture, arise from physical interference on the movement of CSF producing mechanical compression of the brain parenchyma (*direct* mechanism) and the inflammatory reaction, local or far, result from immune-allergic phenomena (*indirect* mechanism) and from the type of response in the host-parasite relationship [2]. Clinical polymorphism characterizes the NCC.

Cerebrovascular manifestations in NCC result from vasculitis, a complication of parenchymal and subarachnoid cysticercosis due to inflammation of small- and medium-sized wall arteries (arteritis) or vein (phlebitis), and occur in 2 to 15% of cases [3]. Vascular reaction is a common finding, has variables responses in the intensity of inflammatory response, and is not related to the intensity of changes in the vessel wall. The clinical manifestation can be either headache migraine-like, ischemic cerebral infarction of variable extensions, hemiplegia, and lacunar syndrome [3–6]. Infarction is more commonly seen with the involvement of the basal cisterns and during the inflammatory stages, being determined by the extent of arachnoiditis. It also can evolve without clinical evidence of cerebral ischemia [7]. Subarachnoid haemorrhage due to mycotic aneurysm, thalamomesencephalic syndrome, parenchymal and intracystic haemorrhage, cerebral haemorrhage, and nominal aphasia are very rare complications [3, 8, 9].

“Berry” or saccular aneurysms are round or saccular dilations. 80–90% of cases are characteristically found at arterial bifurcations in the circle of Willis and in their major branches or connections. They are more common in women than in men (3 : 2) during the 50th to 70th decades and are multiples in 15–20% of patients. The ethiology and pathogenesis remains controversial, although considerable evidences exist to be a multifactorial participation. Inflammatory aneurysms, also denominated mycotic and infectious aneurysms, are fusiform (spindle-shaped) dilations of arterial branches far from arterial bifurcation in 55% of middle cerebral artery and anterior cerebral artery, constitute 2–6% of the aneurysms in adults and 10% in children, and are generally multiples. Inflammatory aneurysms may be of intravascular origin from an extension of an adjacent or a distant focus of bacterial, fungal, or tuberculous infection [10, 11].

In the literature, four patients with NCC and aneurysm were related [9, 12–14]. Our objective is to report a case of a casual association of aneurysm and NCC.

## 2. Case Report

**Figure 1:**
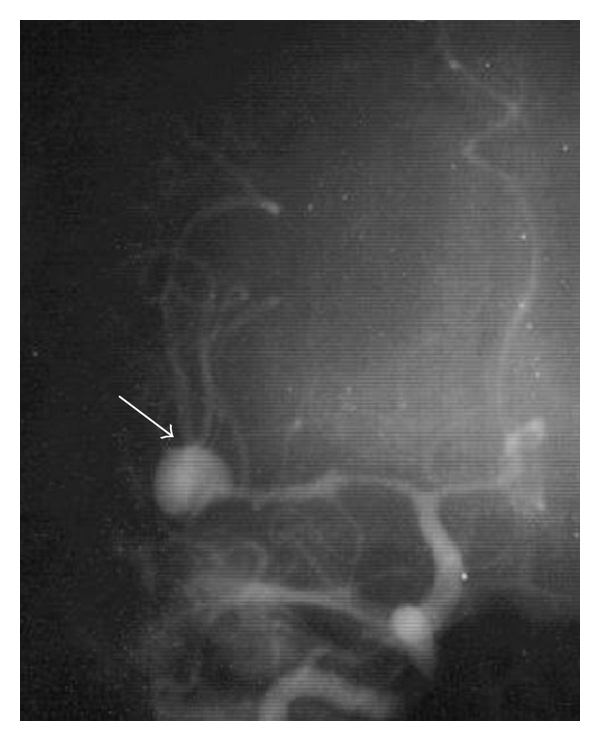
Anteroposterior view of the right carotid angiography showing an aneurysm (arrow) at the bifurcation of the right middle cerebral artery.

**Figure 2:**
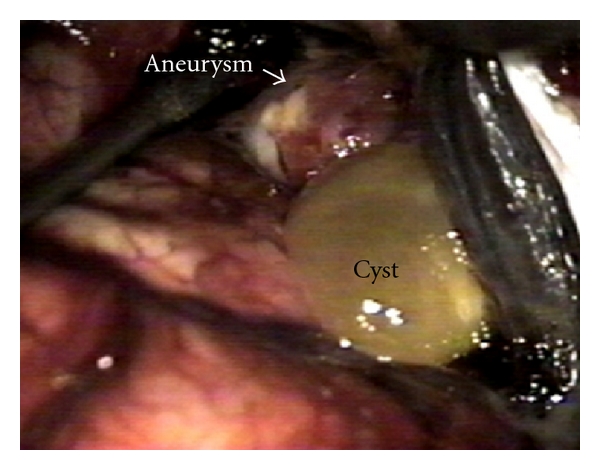
Operative photography of the cyst and the aneurysm.

**Figure 3:**
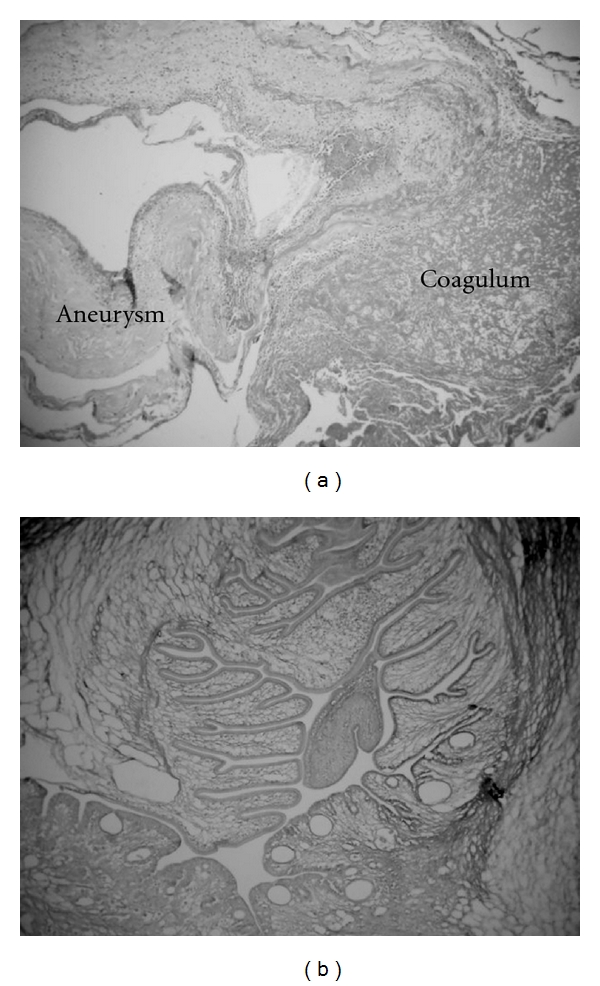
(a) Histopathology of the aneurysm showing a coagulum near the rupture site without signs of inflammation (hematoxylin-eosin stain, original magnification x 6). (b) Histopathology of the excised cyst demonstrating its pathognomonic features of a viable cysticercus without signs of degeneration (Calleja's stain, original magnification x 6).

**Figure 4:**
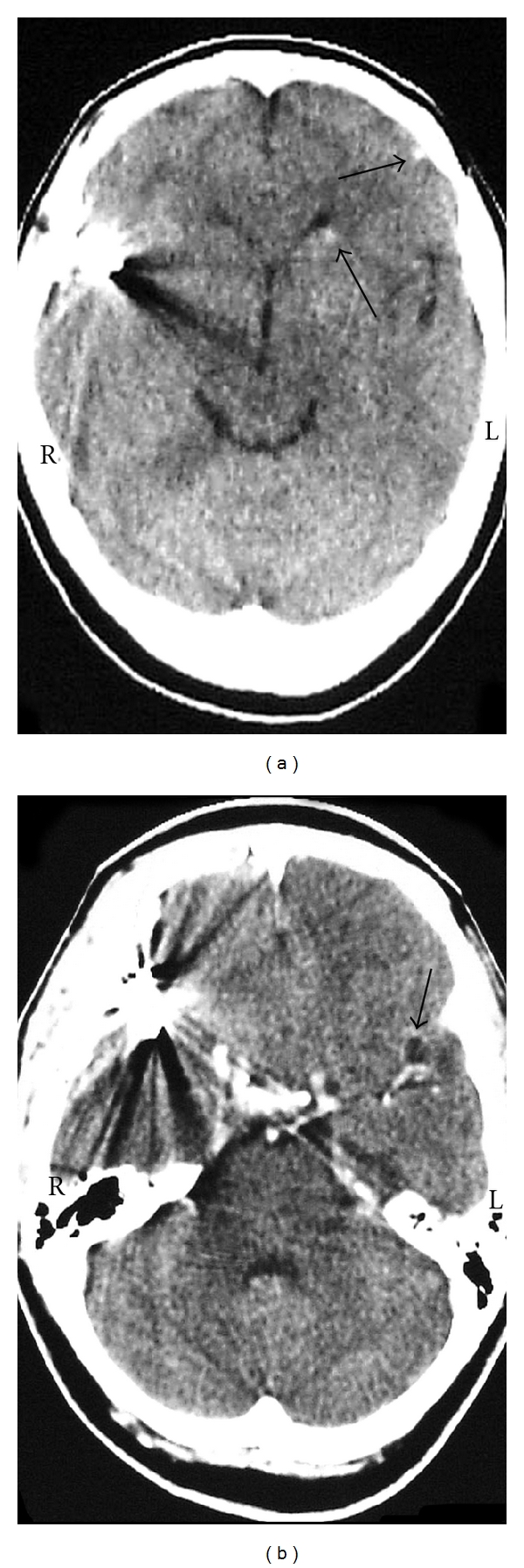
**(**a) CT scan with no contrast after surgery showing calcifications (arrows). (b) Cyst (arrow) enhanced after contrast. (R: right, L: left).

A 49-year-old woman presented an abrupt onset of bilateral frontotemporal headache with vomiting, followed by generalized crisis. It was not possible to conduct computerized tomography (CT). The lumbar puncture confirmed subarachnoid haemorrhage. The angiography (Figure 1) showed an aneurysm at the right middle cerebral artery bifurcation. A right pterional approach to clip the aneurysm was performed. During surgery (Figure 2), a 2 cm of diameter cyst with clots and citrine liquid, suggestive of cysticercus was found in the distal portion of the aneurysm. Dissection and extirpation of the cyst were performed and followed by resection of the aneurysm body after its clipping. Their histopathology proved to be a whole cysticercus (Figure 3(a)) and a broken aneurysm with a coagulum in formation (Figure 3(b)). Postoperative CTs showed multiple calcifications compatible with cysticercosis (Figure 4(a)) and cyst in the left Sylvian cistern, with discrete contrast retention (Figure 4(b)). The patient received treatment with albendazole (15 mg/kg/day during 30 days) associated to dextrochlorpheniramine (12 mg/day). She released from the hospital 21 days after admission, without complains and using anticonvulant and antihypertensive drugs. Three years after surgery she abandoned the followup.

## 3. Discussion

The association of inflammatory aneurysm with cysticercosis is certainly an unusual event. Four cases (Table 1) have been reported on subarachnoid haemorrhage due to inflammatory aneurysm related to NCC [9, 12–14]. On pathological examination, in all previous reports, the cyst was diagnosed as a cysticercal cyst. The presence of inflammatory infiltrate and adhesions surrounding the parasite suggests that inflammation may play an important role in their genesis. In three cases, the manifestation of subarachnoid haemorrhage resulted from vascular alterations caused by vasculitis or by the presence of a pre-existing aneurysm, and occurred with already in degeneration cysts [9, 12, 13]. But, the exact mechanism of its formation is hypothetical, and a congenital origin cannot be ruled out since it is well known that inflammatory reactions in NCC vary from patient to patient [14].

On pathological examination, in all previous reports, the cyst was diagnosed as a cysticercal cyst, but in three of them the aneurysm had no histopathology study [9, 12, 13]. The inner location of aneurysm in the area of severe arachnoiditis around a cysticercus suggest inflammatory origin but does not confirm it. In these cases, the presence of inflammatory infiltrate surrounding the parasite suggests that inflammation may play an important role in their genesis. This means that the exact mechanism of its formation is hypothetical, and a congenital origin cannot rule out since it is well known that inflammatory reactions in NCC vary from patient to patient. The aneurysm's location as well as the presence of inflammatory signs and adhesions supports a suspicion of inflammatory aneurysm, only corroborated by Kim's report [14].

Our paper present the case of a typical congenital aneurysm (woman, 49 years old, saccular aneurysm located at bifurcation, histopathology confirmation) associated with the presence of cyst but with no signs of acute inflammatory process or adhesions in the surgical field. In this paper, we can affirm that the aneurysm is only a casual relationship with NCC. On the other hand, Kim's case report [14] shows a typical inflammatory aneurysm (man, 69 years old, fusiform aneurysm located at distal branch, histopathology confirmation) and enables to affirm that the aneurysm showed a causal relationship with NCC, although the patient's age was not typical for this type of aneurysm. Kim's case and our case showed the presence of cyst and no signs of acute inflammatory process or adhesions in the surgical field.

We can conclude that in NCC the presence of aneurysm may be consequence as well as finding. Even without an exact definition of aneurysm formation physiopathogenesis, all patients must receive cysticidal treatment in the postoperative period of the aneurysm surgery.

## Figures and Tables

**Table 1:** Characteristics of case report of aneurysm in patients with neurocysticercosis.

Sex	Age (years)	Aneurysm location	Surgical procedures	Surgical field	Microscopy of aneurysm	Report	Year
M	23	Distal branch right MCA	Clipping of proximal artery	Temporal lobe hematoma	N.P.	Zee et al. [12]	1980

M	32	Branch right AICA	Wrapping	Thickening leptomeninges Multiple adhesions Partially necrotic cyst	N.P.	Soto-Hernandez et al. [13]	1996

M	32	M2 branch left MCA	Clipping	Inflammatory changes Extensive adhesions Multiple degenerated cysts	N.P.	Huang et al. [9]	2000

M	69	Distal branch right ATA	Trapping	Intracerebral hematoma Brain swelling Cysternal hemorrhage Yellowish cyst	Inflammatory	Kim et al. [14]	2005

F	49	Bifurcation right MCA	Clipping	No inflammation signs or adhesions Yellowish cyst	Congenital	Agapejev et al. (this paper)	

F: female, M: male, MCA: middle cerebral artery, AICA: anterior inferior cerebellar artery, ATA: anterior temporal artery, N.P: not performed.
